# Technical and operational underpinnings of malaria elimination from Sri Lanka

**DOI:** 10.1186/s12936-019-2886-8

**Published:** 2019-07-29

**Authors:** Risintha Premaratne, Rajitha Wickremasinghe, Dewanee Ranaweera, W. M. Kumudu T. de A. W. Gunasekera, Mihirini Hevawitharana, Lalanthika Pieris, Deepika Fernando, Kamini Mendis

**Affiliations:** 1 Anti Malaria Campaign, 555/5 Public Health Building, Narahenpita, Sri Lanka; 2 Department of Public Health, Faculty of Medicine, University of Kelaniya, P.O. Box 6, Thalagolla Road, Ragama, Sri Lanka; 3 Regional Malaria Office, Hambantota, Sri Lanka; 4 Department of Parasitology, Faculty of Medicine, University of Colombo, 25 Kynsey Road, Colombo, Sri Lanka; 5 Colombo 5, Sri Lanka; 6 Present Address: World Health Organization Regional Office for South East Asia, New Delhi, India

**Keywords:** Malaria elimination, Sri Lanka, Entomological surveillance, Case surveillance, Anti-relapse treatment

## Abstract

Malaria was eliminated from Sri Lanka in 2012, and the country received WHO-certification in 2016. The objective of this paper is to describe the epidemiology of malaria elimination in Sri Lanka, and the key technical and operational features of the elimination effort, which may have been central to achieving the goal, even prior to schedule, and despite an ongoing war in parts of the country. Analysis of information and data from the Anti Malaria Campaign (AMC) of Sri Lanka during and before the elimination phase, and the experiences of the author(s) who directed and/or implemented the elimination programme or supported it form the basis of this paper. The key epidemiological features of malaria on the path to elimination included a steady reduction of case incidence from 1999 onwards, and the simultaneous elimination of both *Plasmodium falciparum* and *Plasmodium vivax*. Against the backdrop of a good health infrastructure the AMC, a specialized programme within the Ministry of Health operated through a decentralized provincial health system to implement accepted strategies for the elimination of malaria. Careful planning combined with expertise on malaria control at the Central level with dedicated staff at all levels at the Centre and on the ground in all districts, for several years, was the foundation of this success. The stringent implementation of anti-relapse treatment for *P. vivax* through a strong collaboration with the military in whose cadres most of the malaria cases were clustered in the last few years of transmission would have supported the relatively rapid elimination of *P. vivax.* A robust case and entomological surveillance and investigation system described here enabled a highly focused approach to delivering interventions leading to the interruption of transmission.

## Historical background

The elimination of malaria from Sri Lanka in 2012 and certification by the World Health Organization (WHO) as ‘malaria-free’ in 2016 are historic landmarks for a country, which endured endemic malaria for centuries past [1]. The speckled malaria history of Sri Lanka includes a massive epidemic in 1934/35 in which over 1.5 million cases and 80,000 deaths were recorded [2]. It also includes a near elimination effort in 1963 as part of WHO Global Malaria Eradication Programme in which the malaria incidence was reduced to a mere 17 cases [3]. With the reduction of cases, financial and political commitment to eliminate the disease faltered leading to a resurgence of malaria, which lasted for the next 5 decades. Following this failed attempt at elimination, Sri Lanka adopted a malaria control policy driven by indoor residual spraying (IRS) and treatment of cases with chloroquine and primaquine [3].

The next 4 decades saw the typical endemic pattern of unstable malaria in Sri Lanka, with cycles of epidemics every 10 years or so, overshadowing the annual seasonal fluctuations in incidence [3]. Against a backdrop of sound malaria control efforts, it was since 1999 that a steady decline of malaria incidence occurred until zero indigenous cases were reported from November 2012 onwards. This paper reports the nature of declining malaria and describes the key interventions that were applied during the period that led to the elimination of malaria. The health infrastructure in the country and the operational environment as the backdrop against which the elimination programme was carried out is also outlined. The effort was all the more significant given that a separatist war raged in the country during the period 1983 to 2009, mainly in the northern and eastern provinces both of which were endemic for malaria [4].

### The nature of declining malaria

**Fig. 1:**
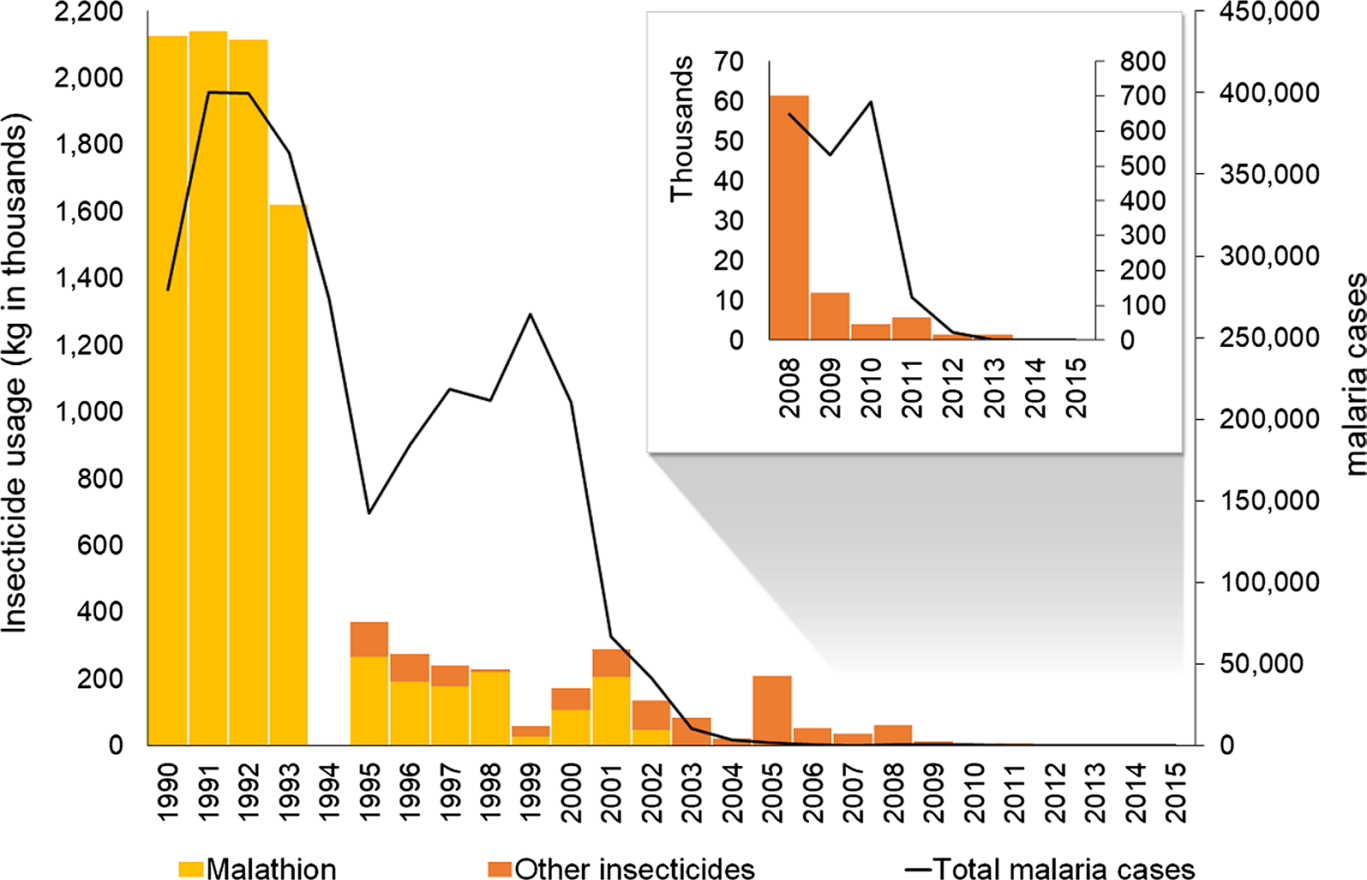
Reported malaria cases and insecticide usage by AMC, 1990–2015. In inset data for the period 2008 to 2015 at a higher magnification (Data for 1994 was not available)

From 1999 onwards, coinciding with the Global Roll Back Malaria Initiative launched by the WHO, malaria control activities in Sri Lanka experienced a revival and were implemented with a renewed determination. Intensified parasitological surveillance focusing on early diagnosis and prompt treatment, entomological surveillance, selective vector control, enhanced health awareness and community engagement programmes were carried out by the Anti Malaria Campaign (AMC), leading to a significant reduction of malaria cases in the country from 1999 onwards. A 68% reduction in incidence was recorded between the year 2000 and 2001. The further decline in the incidence continued in subsequent years, with a 38% decrease recorded from 2001 to 2002 and a 75% decrease recorded in the following year [5] (Fig. 1). In 2002, the AMC received its first grant for malaria control and elimination from the Global Fund to fight AIDS, TB and Malaria (GFATM). The malaria incidence in Sri Lanka had reached pre-elimination levels by 2004 with a caseload of less than 1 per 1000 population. Yet, due to the ongoing separatist war in the north and east of the country at the time which made it difficult to deliver interventions, the AMC held back on a decision to enter into an elimination phase. In 2008, Sri Lanka embarked on the malaria pre-elimination phase with the stated objective of interrupting *P. falciparum* transmission by end of 2012 and *P. vivax* transmission by end of 2014. Case-based surveillance and response was initiated in 2008 since when every malaria case was investigated. The separatist war which made it difficult to deliver interventions, ended in May 2009 and this paved the way for an unhindered effort to eliminate malaria. Intensified surveillance and response led to a further reduction in the number of malaria cases with 124 cases being reported in 2011, which was when the country moved into the malaria elimination phase. The last indigenous case of malaria was reported in October 2012 ahead of the established targets, with 23 cases being recorded earlier that year. Sri Lanka has maintained zero cases of indigenous malaria to date [5]. However, because the risk of re-introduction to, and re-establishment of malaria in Sri Lanka remains high after elimination, intensive surveillance and response measures are being taken to mitigate this threat. The first introduced case of malaria was reported in the country in 2018 [6], 6 years after elimination. Yet, further onward transmission from this case was prevented due to the effectiveness of the current surveillance and response system.

**Fig. 2:**
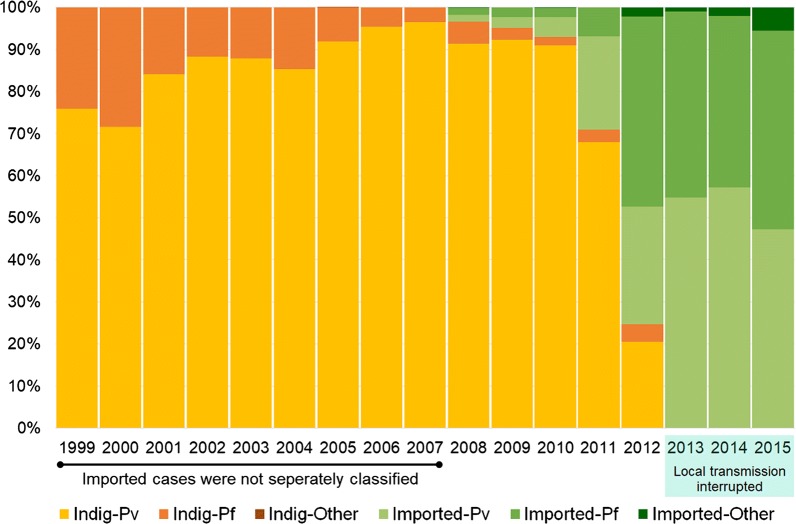
The relative proportions of *Plasmodium vivax* and *Plasmodium falciparum* in indigenous and imported malaria infections, 1999–2015. Infections were classified as imported from 2008 onwards. From 2013 onwards there were no local cases

It was from 2008 onwards that the AMC began classifying cases as indigenous and imported [7]. With the reduction of indigenous malaria cases, the absolute number as well as the relative proportion of imported malaria cases increased (Fig. 2) there having been 403 imported cases reported during the period 2008–2015. One of the likely reasons being that with the cessation of the war in 2009, a massive national development drive was launched by the government extending throughout the country including in previously war affected areas in the North and East, which were endemic for malaria. With it there was a surge in the influx of foreign labour from India and China, and tourists from other countries as well. This was a setback to the elimination effort due to the high vulnerability it created, which, when combined with the prevailing receptivity in the country increased the risk for malaria resurgence.

As the malaria incidence dropped the proportion of indigenous *P. vivax* cases increased relative to *P. falciparum* (Fig. 2) although, both species were prevalent till the cessation of transmission. Towards the latter years of the elimination programme malaria infections moved markedly into the adult male population. Whereas every year from 1999 to 2001, 54% or more of cases were in adult males and this proportion gradually increased until the last 3 years before elimination from 2009 onwards when more than 96%, 87% and 93%, respectively, were in adult males Towards the last years before elimination a very large proportion of indigenous cases were in armed forces personnel who served in forested areas of the war-torn regions.

### Health infrastructure and supportive mechanisms for malaria elimination

**Fig. 3:**
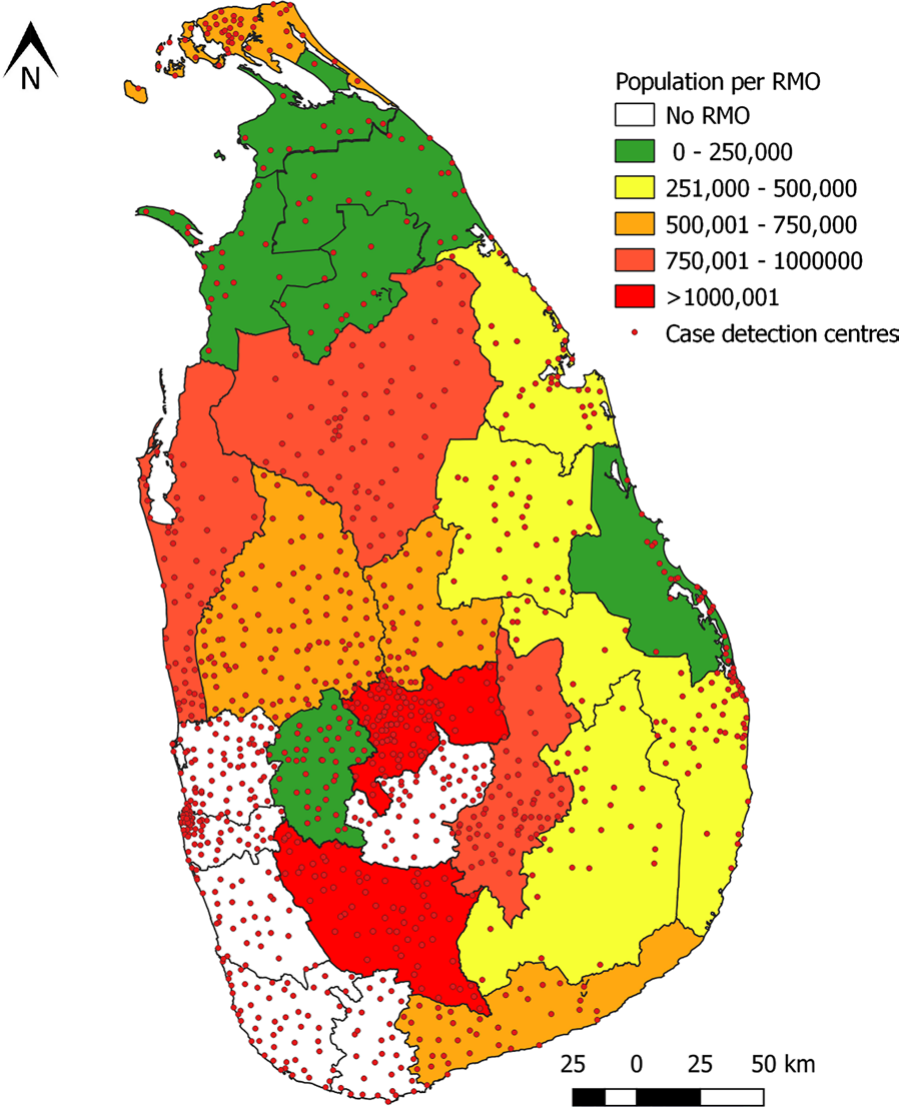
Map of Sri Lanka showing districts, which were served by Regional Malaria Officers. Districts are colour coded on the basis of population per RMO. Districts depicted in white had no RMOs since they were not endemic for malaria. The red dots represent Government health institutions which served as diagnosis and treatment centres for malaria

The Anti Malaria Campaign within the Ministry of Health and Indigenous Medicine (MoHNIM) is responsible for national policy and strategy, and technical guidance for implementation and monitoring and evaluation. In the decentralized health system, malaria control and elimination activities are carried out by the Regional (district) health authorities under the Provincial Ministries of Health. The responsibility for malaria control activities lie with the Regional Malaria Officers (RMOs) who are accountable to the Regional Director of Health Services under the Provincial Health Ministries, but take technical guidance from and report to the central AMC headquarters. RMOs were the coordinators and implementers of the malaria control and elimination programme on the ground. Twenty-two RMOs, many of them highly experienced and dedicated, together with their teams implemented the elimination programme in their regions of the country as depicted in Fig. 3. Each RMO has an office in the Regional Directorate of Health and leads a team comprising Public Health Inspectors, Public Health Laboratory Technicians, Public Health Field Officers, and an entomology team for vector surveillance and vector control. Once a month the AMC convened a meeting of all RMOs and the AMC headquarters staff to report on and review the malaria situation and discuss in-depth the programme of work in the country and to plan further.

An independent group of experts served as a Technical Support Group (TSG) to guide and advice the AMC on technical aspects of malaria elimination. The TSG convened every 2 months under the chairmanship of the Director General of Health Services. In 2014, a subcommittee of this group was appointed as the Case Review Committee which independently assessed every malaria patient and reviewed the case classification made by the AMC [8].

### Elimination strategies

Sri Lanka’s national strategy for malaria elimination was based, broadly, on WHO guidelines for elimination [9], and it included targeted vector control, intensified case surveillance and radical treatment, and case investigation and response. Because the malaria elimination efforts began during the separatist war in the country, the elimination plan was strategically phased out focusing first on the districts where there were no hostilities (Southern, Western and Central regions), then on the transitional zones (Eastern) and finally on the districts engaged in war (Northern region).

### Disease surveillance, case management and follow up

The country’s effective health service delivery system, which had high geographical coverage provided good access to malaria diagnosis and treatment throughout the post-eradication era of the 1960s and 1970s. From 1999 onwards the malaria diagnosis and treatment services were further enhanced with mobile malaria clinics serving pockets of high-risk populations and those in remote rural areas. From 2008 onwards when the country embarked on malaria elimination, epidemiological surveillance and response became a high priority activity. This enabled high coverage of services, early diagnosis, prompt treatment and notification of cases, followed by rigorous case investigation and response in the form of case-based preventive measures and follow up.

Parasitological surveillance was through: (1) passive case detection (PCD), where by malaria was detected among patients who on their own initiative sought treatment and were thereby tested for malaria. The location of medical institutions in the country, which served as PCD centres are shown in Fig. 3. In several of these centres a more pro-active approach was taken in screening patients who attend these medical institutions, for malaria; and (2) active case detection (ACD) where high risk population groups, communities or households were screened for malaria irrespective of the presence of symptoms. Towards the end of the elimination effort when the case numbers were very low or zero, the efforts were also directed at preventing the re-introduction of malaria. Thus, pro-active case detection (PACD) [4, 10–12] was carried out at regular intervals amongst high-risk groups. These activities were carried over to the post-elimination phase to prevent malaria re-introduction, such as in asylum seekers from South Asia, fishermen returning from Africa [13] and security forces personnel returning from UN Peace Keeping Missions in malaria endemic countries [14]. Such groups were often identified through a lead provided by a patient detected by PCD—i.e. an index case, in which instance it was termed reactive case detection (RACD).

Microscopy was the main method used for malaria diagnosis throughout the period, and rapid diagnostic tests (RDTs) were used to a much lesser extent. All RDT positive results in both public and private sector health institutions were subjected to confirmation by microscopy. Confirmed malaria patients were treated by the attending physicians according to national treatment guidelines [15] in close consultation with AMC staff. Since 2008, the first-line treatment for *P. falciparum* was artemisinin-based combination therapy (ACT) (using artemether–lumefantrine) combined with a single dose of primaquine as a gametocytocidal, and for *P. vivax*, chloroquine and as an anti-relapse treatment primaquine for 14 days. The Sri Lanka military which reported most malaria cases towards the end of the elimination phase, adopted the strategy of keeping all malaria patients in barracks until treatment with 14 days of primaquine was completed, and only then were the patients permitted to leave camp for home utilizing their quota of sick leave. This was in response to the observation that the incidence of *P. vivax* relapses were high among military personnel possibly due to poor adherence to primaquine. During the elimination phase primaquine as anti-relapse treatment was given to all *P. vivax* cases under supervision to ensure full adherence. From 2008 onwards every *P. falciparum* patient, and from 2014 onwards all malaria patients in the country were hospitalized for at least 3 days until the course of curative treatment was completed. Until January 2015, therapeutic efficacy of medicines was being assessed in patients (according to WHO guidelines) as special studies, but thereafter routinely on every patient [16]. Even when the number of malaria cases in the country dropped below 200, four to five percent of the entire population of the country continued to be examined microscopically for malaria annually, until and after elimination was achieved (Table 1).

**Table 1:** Average annual blood examination rates (ABER) before and through the elimination phase

Year	Total population	Number of blood smears examined	Annual blood examination rate
1999	18,754,185	1,582,111	8.4
2000	18,941,730	1,781,372	9.4
2001	19,131,147	1,353,386	7.1
2002	19,007,000	1,391,386	7.3
2003	19,252,000	1,192,259	6.2
2004	19,502,098	1,198,181	6.1
2005	19,668,000	973,861	5.0
2006	19,886,000	1,076,121	5.4
2007	20,159,641	1,044,115	5.2
2008	20,217,000	1,047,104	5.2
2009	20,450,000	909,632	4.4
2010	20,653,000	1,001,107	4.8
2011	20,653,000	994,546	4.8
2012	20,263,723	948,250	4.7
2013	20,466,352	988,659	4.8
2014	20,623,888	1,069,817	5.2
2015	20,868,762	1,142,466	5.0
2016	21,203,000	1,072,396	5.1

The role of the private health sector in case surveillance and case management changed as elimination neared. With an increasing general trend of people using private health care, until about 2007, a small proportion of malaria patients sought private health care for malaria as they did for any other illness. Possibly because of the extensive health infrastructure in Sri Lanka, it was a premium private sector that operated in the country, in the form of private medical practitioners throughout the country and private hospitals in cities; neither the informal private sector nor the problem of counterfeit medicines were encountered at any time in relation to malaria. Chloroquine and primaquine were available as anti-malarial medicines in the private sector but gradually as the malaria incidence decreased significantly, and the market for anti-malarial medicines diminished, private dispensaries stopped stocking anti-malarial medicines. From 2008 onwards, when ACT was adopted as the first-line medicine for the treatment of *P. falciparum*, it was only the MoHNIM that was authorized to purchase, stock and distribute them through the AMC. Thus, with the AMC embarking on pre-elimination from 2008 onwards the private sector conformed strictly to case notification of malaria when they diagnosed a case, and the AMC and RMOs provided the anti-malarial medicine to them on a case by case basis upon being notified. Since a proportion of patients continued to be diagnosed with malaria in the private sector, especially imported malaria cases, the AMC provided guidance on quality RDT products to be procured, and training to microscopists in private hospitals free-of-charge on a regular basis, a practice that continues to date.

Several standard measures were taken for quality assurance and quality control of malaria microscopy during the control phase, such as cross-checking of all positives and 5% of negative blood smears which has been the standard for maintaining the quality of malaria microscopy. Since 1998, In-service training programmes were conducted annually for laboratory technicians performing malaria microscopy. To establish a “core group of expert malaria microscopists” the AMC in collaboration with the Asian Collaborative Training Network for Malaria (ACTMalaria) conducted training for microscopists and proficiency assessment in 2014 and 2016. Reference slides were provided to microscopists and refresher training was conducted based on the performance of panel testing [5]. It was, however, not until 2017 that Sri Lanka entered into the WHO’s regional programme for accreditation of malaria microscopists. The quality of RDTs was ensured by procuring WHO prequalified products.

**Fig. 4:**
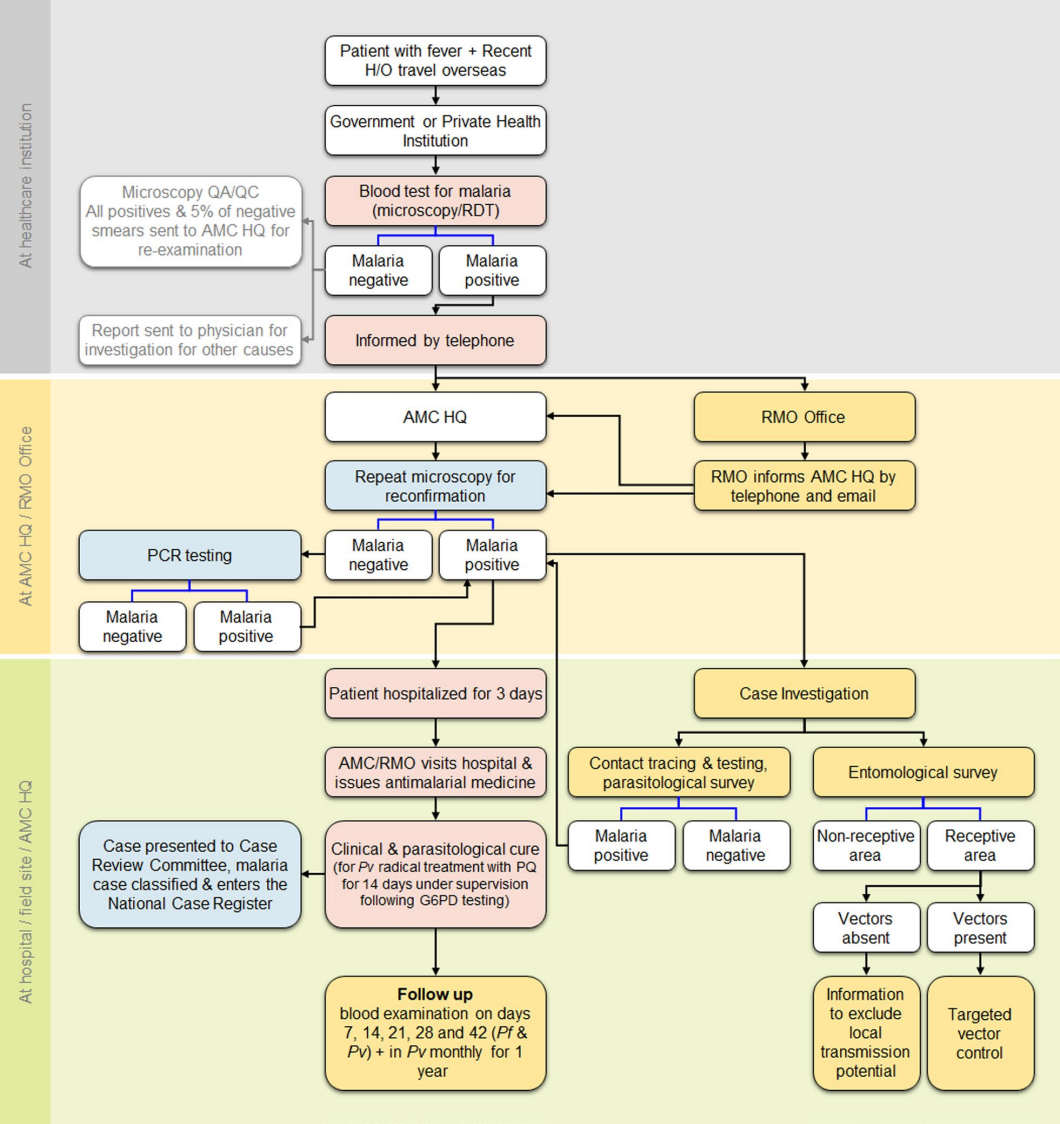
Sequence of events that follow when a suspected malaria patient presents at a health institution in either the public private sector. The boxes shaded in colours represent actions, in pink at the health care institutions; in blue at the AMC headquarters and in yellow at the RMO offices. Boxes in white represent outcomes

When the diagnosis of malaria was confirmed in either the public or the private sector, the case was notified within the next 24 h, and a case investigation was begun within 48 h of detection. The sequence of events that followed when a suspected malaria patient presents at a health institution is provided in Fig. 4. The response to the case commenced within 72 h of detection and included RACD by screening householders and local contacts residing within approximately 1-km radius of the index case’s residence or wherever the index case had stayed overnight during the 2 weeks preceding the onset of symptoms. In addition to the persons living in the same locality as the case, people who had a similar exposure in the same environment in which the index case was suspected to have acquired malaria overseas (i.e. pilgrims from the same travel group and occupational groups visiting malaria endemic countries) were traced and tested for malaria. Contact tracing and screening proved to be extremely important during the malaria elimination phase in Sri Lanka. These individuals may be found within the same localities in which the index case resides or may be scattered in other parts of the country. Coordination and collaboration between the RMOs of different districts ensured that all individuals who came in contact with the index case were screened, enabling the capture of infected persons who had the same exposure to malaria as the index case, who might have been otherwise diagnosed later or even overlooked. The flow of information on cases, entomology and responses was maintained by either mobile or land telephone and using electronic communication means, because a fully functional web-based information system was not in place during the elimination effort.

Follow up of confirmed malaria cases by repeated microscopic examination of blood was performed on days 7, 14, 21, 28, 42 and monthly for 1 year in *P. vivax* and *Plasmodium ovale* infections (considering the date of diagnosis as day 0) and days 7, 14, 21, 28 and 42 for *P. falciparum* infections. Every malaria case reported in Sri Lanka since 2011 has thus been investigated and followed up for the required period of time, unless the patient left the country during the follow-up period.

Chemoprophylaxis was recommended for travellers to malaria endemic countries who are at risk of acquiring malaria such as UN Peace Keeping Forces, gem traders who travel to African countries and stayed for weeks at gem mining sites, safari travellers to Africa, and middle level businessmen travelling to South India. They were provided with malaria prophylactic medicines free-of-charge by AMC.

The supply chain for medicines and diagnostics ensured that there were no stock-outs of either commodity at any of the stocking points. The stocks of malaria commodities (medicines and diagnostics) were maintained at service delivery points such that if a first or second-line medicine or any other commodity was required for malaria case management at any service delivery point at which it is not stocked, it can and will be delivered within a few hours.

### Entomological surveillance and vector control

**Fig. 5:**
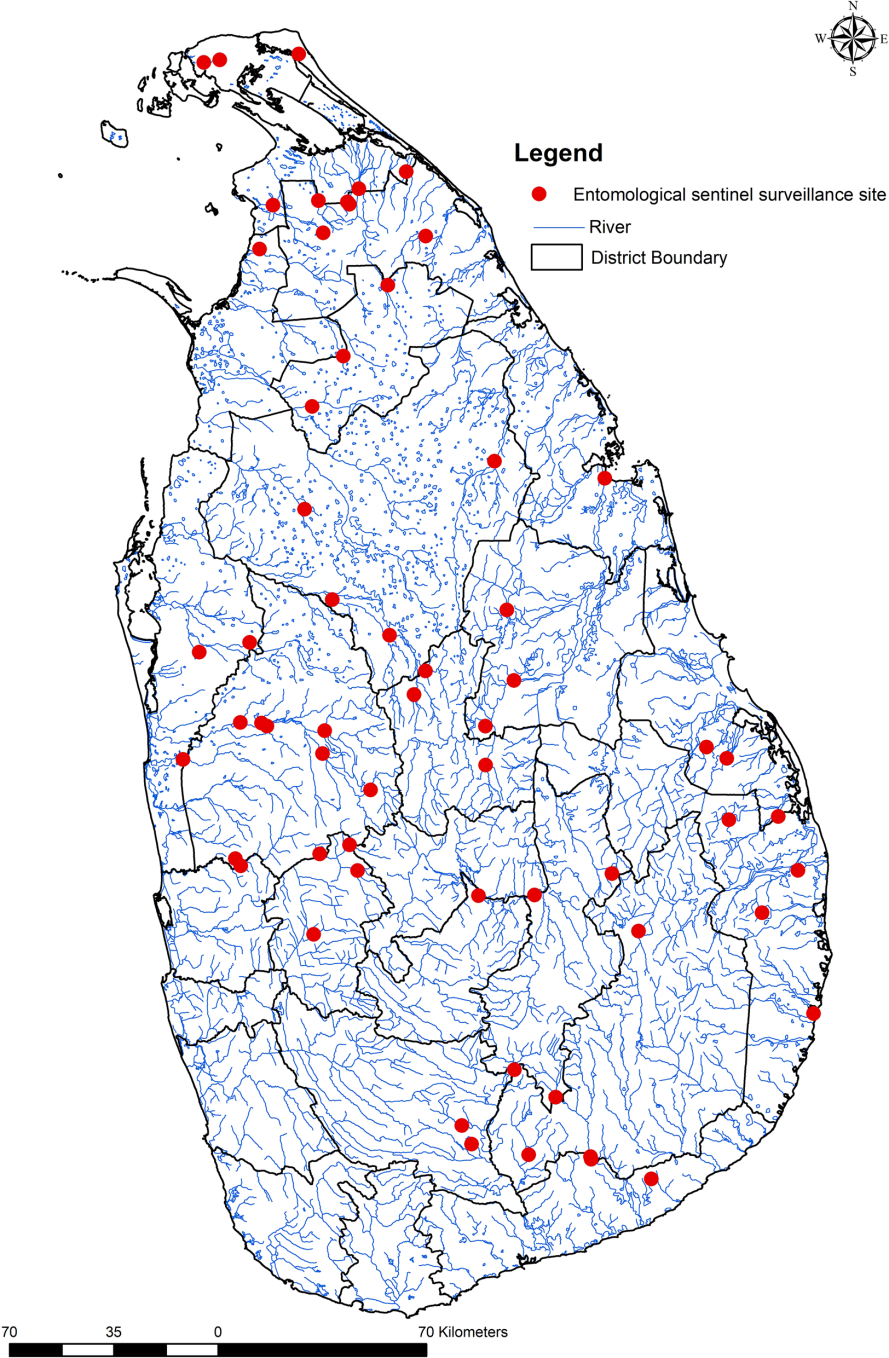
Map of Sri Lanka showing entomological surveillance sites. Circles represent entomological surveillance sites. District boundaries are in black lines and major waterways are represented by blue lines

During the elimination phase, *Anopheles culicifacies,* the primary malaria vector in Sri Lanka, was prevalent in the country as it was during the control phase. From 1999 onwards entomological surveillance was intensified. Most RMOs maintained a minimum of two sentinel sites in their region for conducting surveillance every year and thus over 50 sentinel sites were maintained in the country (Fig. 5). Additionally, spot surveillance checks were carried out in other areas to determine receptivity to control vectors if and when necessary. These surveys were carried out when there were changes either in environmental conditions making them favourable for vector breeding (e.g. climatic changes, natural disasters) or anthropogenic activities such as those related to development projects and gem mining, or in the vulnerability of populations (e.g. where resettled communities, migratory populations and foreign workers were present).

Case based entomological surveillance commenced very early in the elimination phase to evaluate the receptivity of the area by determining the presence of vectors. This was initiated within 48 h of notifying a malaria case, many of which were imported. This was a reactive survey carried out covering a distance of approximately 1-km radius from the location of the case. A comprehensive larval survey was carried out initially to investigate the presence of potential breeding habitats in the area. Adult surveys were also carried out routinely. A wide range of surveillance techniques were used i.e. cattle baited hut collections, cattle baited trap collections, larval surveys to determine vector prevalence, indoor resting and outdoor resting aspirator collections, pyrethrum spray sheet collections, window traps to determine resting behaviour, human landing catches both partial and full night to determine biting behaviour, and larval surveys to assess the breeding behaviour. They were used in combination to gather information on receptivity of an area. Whenever a high receptivity was found by entomological surveillance together with a high vulnerability due to the presence of risk groups, appropriate vector control measures were implemented. The efficacy of insecticides used for IRS and long-lasting insecticidal nets (LLINs) on mosquito populations was assessed by regularly carrying out insecticide susceptibility tests and bioassay tests, respectively.

The malaria control programme in Sri Lanka relied heavily on blanket IRS coverage in endemic areas for vector control during the period, which followed the late 1960s but moved to more focally targeted application of IRS in the late 1990s, together with implementation of integrated vector management strategies. These vector control activities were implemented in previously malarious areas or malaria foci, areas with a relatively high incidence of *P. falciparum* infections, hard- to-reach areas and in areas in which vulnerable populations, including internally displaced people were located. IRS was gradually tapered off from 2001 onwards (Fig. 1). The decline in IRS coverage closely corresponds with the decline in the number of reported cases throughout the country.

Long-lasting insecticide-treated nets were introduced in 2002 to high-risk areas. In addition to the LLINs distributed by the AMC, several other organizations that were assisting internally displaced persons during this time also distributed LLINs as part of their assistance package.

The AMC also initiated larviciding programmes using locally available larvivorous fish. Stock tanks were built in selected RMO complexes for rearing *Poecilia reticulata* [17–19]. These fish were then introduced through non-governmental organizations and school health clubs in selected sites where vector breeding was demonstrated and conducive for introduction of larvivorous fish. Chemical larviciding using temephos was carried out in locations where larvivorous fish could not be introduced. These sites included downstream from major dams where pooling frequently occurred and other temporary water collections such as abandoned gem pits that are favoured sites for mosquito breeding, which had resulted in many documented outbreaks [20]. The AMC filled these pits in mining areas. Where waterways were dammed, intermittent flushing of the canals and waterways were carried out as a part of malaria control activities among settler farmer families. These were part of the integrated vector management (IVM) strategy adopted at that time.

During the pre- and elimination phases, from 2009 to 2014 the AMC was partnered for implementation by two other principal recipients of the Round 8 Global Fund Grant. One was Sarvodaya, a community based non-governmental organization (NGO) of high repute in Sri Lanka and the other was Tropical and Environmental Diseases and Health Associates (TEDHA), a private sector organization with a team of malaria experts. Many other inter-sectoral partnerships and collaborations of the AMC with several departments, organizations and agencies were critical to the success of elimination programme [5].

## Discussion

It is evident that Sri Lanka’s elimination effort was founded on a background of good malaria control, that was reinforced greatly from 1999 onwards by a focused and targeted approach to achieving universal coverage for access to diagnosis and treatment and vector control. This approach led, as expected, to a steady decrease in malaria incidence. The opportunity was seized by the AMC leadership in 2008 to switch to a pre-elimination/elimination phase by adopting, and extending to the entire country a heightened surveillance system which enabled an even more focused application of interventions until the last case of indigenous malaria was dealt with.

Whilst adopting and implementing standard WHO recommended policies and strategies for malaria elimination there were a few aspects of the programme, which is believed to stand out as having been critically important to achieving the interruption of transmission ahead of schedule. One of the most important was the human resource capacity on the ground and the committed leadership at the AMC headquarters. RMOs were highly experienced and dedicated professionals with malaria expertise, each of whom had a geographical area of about 3000 km^2^ and an average population of 670,000 in which to plan and implement an evidence-based elimination programme. Their knowledge of the geography, risk areas, population groups and their movements, and potential vector breeding sites was essential for Sri Lanka’s highly focused and targeted approach to elimination. The mobile malaria clinics which supplemented the existing health care delivery system for malaria was thus directed by RMOs to appropriate places—those at high risk because of vector prevalence, or due to poor access of populations to diagnosis, or any other focused local event or occurrence which increased the risk for transmission [3]. RMOs also acted with discretion in real time, on local data, which they generated through the strong surveillance system for both parasites and vectors. RMOs were well connected to each other, which enabled action to be taken on population movement. The monthly review meetings at AMC ensured a highly coordinated effort countrywide. During the ongoing separatist war, it was the diligence and commitment of RMOs, including, and especially, those in the war affected areas, combined with technical guidance and supervision from AMC headquarters, and an uninterrupted supply of malaria commodities, which the AMC maintained to all districts, which made the steady progress in elimination possible. Based on this experience, it can be concluded that competent people on the ground in sufficient numbers that allows each to deal with a manageable geographical area is a vital requirement for malaria elimination. They need to have a good knowledge of the geo-spatial factors relating to malaria and be supported by a team of workers that would enable them to act on local data in real time. This may be of particular relevance to large countries targeting malaria elimination, where the ratio of competent malaria control staff to geographical areas and populations tend to be very low because administrative units are large areas often with large populations.

Sri Lanka’s sound health infrastructure and comprehensive health care delivery system coupled with an extensive road network, and a high literacy rate among its people obviously made the elimination of malaria, as it would do any public health achievement, more feasible. An uninterrupted supply chain of commodities that was sustained and a reasonable effort at quality control of products and services were supportive elements. Almost anywhere in the country people had access to diagnosis and treatment for malaria within two hours of developing symptoms. Introducing, in 2005, proactive and reactive case detection following case investigation, over and above the passive detection system was an important component of the accelerated elimination programme. The annual blood examination rate (ABER) is an index which provides some measure of the adequacy of a case surveillance system. The ABER of 4–5% which was maintained in the country as a whole, falls well within the range of ABERs that WHO recommends should be maintained during the elimination phase [21]. Furthermore, because malaria screening was carried out mainly on population groups who were at risk of malaria, it is likely that the ABER would have been much higher in populations that mattered. Towards the end of the elimination programme the country’s efforts were focused as much on preventing the re-introduction of malaria through chemo-prevention of travellers to endemic countries, heightened ACD, and public awareness programmes, as on mopping up the residual infections.

The separatist war which raged in the northern and eastern provinces from 1983 to 2009 was the most critical challenge to malaria control and later to elimination. The war was not continuous in time or space, and the warring years were interrupted by periodic attempts at peace negotiations during which there was temporary cessation of fighting creating intervals which allowed public servants to maintain services. Even in the war zones, there was cooperation between the MoHNIM and the warring factions on malaria control and other essential health programmes such as immunization, with the parties even declaring cease-fire days to enable such programmes to function. The MoHNIM provided uninterrupted supplies of malaria commodities to rebel held areas in order that people had access to them. Where there were gaps in service delivery by the government, several local and foreign NGOs and international organizations which functioned in the war affected areas provided services. The RMOs of war affected areas and their teams remained in their posts throughout the period of war at their own will, and functioned within the boundaries that allowed them to. With hindsight, equity in the distribution of preventive and curative services relating to malaria must have played an important part in achieving malaria elimination. Exclusion of any population group from accessing these services as happens in some malaria situations in the world, either because of outreach, wars, or any form of discrimination will lead to a persisting parasite reservoir, and thus severely compromise efforts to eliminate malaria.

*Plasmodium vivax* was by far the predominant species of malaria as transmission decreased and most infections were among soldiers who fought the war. They moved in forested areas mostly at night (coinciding with peak time for vector bite), which increased the risk of infectious bites. It is notable that the elimination of *P. vivax* was achieved well ahead of the established targets, and simultaneously with *P. falciparum*, a phenomenon rarely observed in malaria elimination experiences of other countries [22]. Owing to its biological properties, which confer a higher survivability [23], endemic *P. vivax* usually lingers on for several years after the elimination of *P. falciparum* [24]. The elimination of *P. vivax* simultaneously with *P. falciparum* in Sri Lanka was largely, if not entirely attributable to the rigorous implementation of radical treatment for *P. vivax* infections that was made possible by the close collaboration of the AMC with the military. Had it not been for this collaboration the movement of soldiers from the war zones to their homes in various parts of the country, whilst they harboured dormant liver stage parasites may well have undermined the entire elimination effort in Sri Lanka. This, in fact, was the phenomenon underlying the resurgence of *P. vivax* malaria in the Republic of Korea many years after malaria elimination—soldiers serving at the border in the demilitarized zone carried infections to other parts of the country leading ultimately to the re-establishment of *P. vivax* malaria which still prevails in the country [25, 26].

Entomological surveillance played a very prominent role in Sri Lanka’s elimination effort to the extent that questions were raised about its cost effectiveness as an intervention. The background to this comprehensive entomological surveillance programme was that the AMC had, traditionally, a strong entomology expertise in the central headquarters as well as within the RMOs, and a comprehensive evidence-base was available on the state of malaria vectors in the country. With hindsight, the rigorous entomology surveillance programme is what enabled a highly targeted and situation-specific vector control programme to be implemented, which served the elimination effort extremely well. It allowed not only the tapering off of IRS and the moderate use of LLINs, but also an effective integrated vector management system which included larviciding with chemicals and with larvivorous fish which was used widely and effectively where indicated. Again, the use of such specific locally adapted vector control interventions was only possible owing to the efforts of the RMOs who had a sound understanding of the bio-environments in which they worked and integrated vector control with discretion. This approach could serve as an example for elimination in other areas of unstable malaria, which prevails in most parts of the world outside sub-Saharan Africa.

One of the highest efficiencies of Sri Lanka’s elimination programme was the rapidity with which the case notification, case investigation and responsive action was initiated on days 1, 2 and 3 respectively from the day of detection. This is a further improvement on the 1, 3, 7 approach adopted successfully by China and recommended as a standard by the WHO [27]. It is noteworthy that this was achieved without the support of a state-of-the-art web-based information system. It can be conceded that it was the small size of the country and the strong and disciplined malaria elimination workforce on the ground that made this possible, and that it may not be achievable in all situations. As for structural aspects of the programme, the AMC had a strong element of verticality, but operated very successfully and almost entirely through a decentralized provincial and district health system. With this experience, it is difficult to imagine that a successful elimination programme could be conducted without an element of “verticality” in its structure. Aspects relating to financing of the elimination programme are beyond the scope of this paper.

## Conclusions

The Sri Lankan experience suggests that a sound health infrastructure coupled with a strong, technically sound central level leadership, a high human resource capacity and malaria expertise on the ground were important to achieve elimination. It is concluded that a disciplined programme which responded to ground realities available locally and effective collaborations where needed such as the one with the military, underpinned the success of elimination despite an ongoing war in some parts of the country. The absence of any of these features would make malaria elimination more difficult, though not impossible as suggested by the experiences of other countries in eliminating malaria.

## Data Availability

The datasets generated and/or analysed in this publication are not publicly available due to the fact that they belong to the Ministry of Health, Sri Lanka. Clarifications regarding data can be made through Dr. Risintha Premaratne, former Acting Director of the Anti Malaria Campaign who is an author of this publication.

## Abbreviations

ACD: active case detection
ACTMalaria: Asian Collaborative Training Network for Malaria
AMC: Anti Malaria Campaign
ACT: artemisinin-based combination therapy
ABER: annual blood examination rate
GFATM: global fund to fight AIDS, tuberculosis and malaria
IRS: indoor residual spraying
IVM: integrated vector management
LLIN: long-lasting insecticidal nets
MoHNIM: Ministry of Health, Nutrition and Indigenous Medicine
NGO: non governmental organization
PACD: proactive case detection
PCD: passive case detection
RACD: reactive case detection
RDT: rapid diagnostic tests
RMO: Regional Malaria Officer
TEDHA: Tropical and Environmental Diseases and Health Associates
TSG: Technical Support Group
WHO: World Health Organization
